# How loss-of-function mutations in *IFIH1* contribute to infectious and/or inflammatory disease – a systematic review

**DOI:** 10.1016/j.jtauto.2026.100353

**Published:** 2026-02-04

**Authors:** Isabelle Ince, Francesca Sposito, Amandine Charras, Liza J. McCann, Christian M. Hedrich

**Affiliations:** a Department of Women's and Children's Health, Institute of Life Course and Medical Sciences, University of Liverpool, UK; b Department of Rheumatology, Alder Hey Children's NHS Foundation Trust, Liverpool, UK

**Keywords:** IFIH1, MDA5, Interferon, Inflammation, Disease, Deficiency, Infection

## Abstract

The *IFIH1* gene encodes for the cytoplasmic innate immune receptor *Melanoma Differentiation-Associated protein 5* (MDA5) that detects viral double-stranded RNA to initiate type I interferon (IFN) responses. While gain-of-function mutations in *IFIH1* have been linked with systemic inflammatory diseases, loss-of-function remains less well understood.

This systematic review, following *Preferred Reporting Items for Systematic reviews and Meta-Analyses* (PRISMA) guidance, explored how *IFIH1*/MDA5 loss-of-function affects susceptibility to virus infections and/or contributes to inflammatory diseases.

Sixteen loss-of-function variants affecting *IFIH1* were discussed across 33 studies. Loss-of-function variants were consistently associated with increased susceptibility and/or severity of **virus infections**, including severe acute respiratory syndrome coronavirus (SARS-CoV2) and human immunodeficiency virus (HIV). Several rare biallelic *IFIH1* mutations lead to profound immunodeficiency, while heterozygous mutations associate with milder clinical presentations. Likely through dampening IFN responses, several variants **protect from the development of inflammatory diseases, including** type 1 diabetes and hypothyroidism. However, *IFIH1* deficiency is also implicated in the development of inflammatory diseases, including i**nflammatory bowel disease. Moreover,** the presence of **inactivating** anti-MDA5 antibodies may alter the clinical phenotypes and prognosis of dermatomyositis and infections with SARS-CoV2. Though their exact impact on MDA5 function has not been confirmed experimentally, anti-MDA5 antibodies may result in loss-of-function and impaired host defence against viruses.

Loss of *IFIH1*/MDA5 activity has diverse effects on anti-viral immunity, associated damage and susceptibility to inflammatory disease, but also protection against organ-specific immune-mediated pathology. Findings highlight the importance of *IFIH1* in immune regulation and warrant future studies exploring its potential as a diagnostic and therapeutic target.

## Highlights

• The innate immune receptor MDA5 (encoded by *IFIH1*) impacts on anti-viral host defence and immune homeostasis.
• Loss-of *IFIH1*/MDA5 function associates with infections and autoimmune/inflammatory diseases.
• While reduced MDA5 function limits the risk of autoimmune/inflammatory diseases associated with virus infections, it increases the risk for IBD.
• MDA5 inactivation through autoantibodies affects clinical phenotypes in inflammatory myopathies.

Tight regulation of MDA5 (*IFIH1*) is essential for immune homeostasis and the prevention of tissue damage.

## Abbreviations:

AIDS: – Acquired Immunodeficiency Syndrome
ADAR1: – Adenosine Deaminase Acting on RNA 1
ATP: – Adenosine Triphosphate
CADM: – Clinically Amyopathic Dermatomyositis
CARD: – Caspase Activation and Recruitment Domain
CNS: – Central Nervous System
CTD: – C-Terminal Domain
CVB3: – Coxsackievirus B3
DM: – Dermatomyositis
dsRNA: – Double-Stranded RNA
EV: – Enterovirus
HIV: – Human Immunodeficiency Virus
IBD: – Inflammatory Bowel Disease
IFIH1: – Interferon Induced with Helicase C Domain 1
IFN-I: – Type I Interferon
IL: – Interleukin
ILD: – Interstitial Lung Disease
IRF3/IRF7: – Interferon Regulatory Factor 3/7
IVS: – Intervening Sequence (used in intron position notation)
MAVS: – Mitochondrial Anti-Viral Signalling Protein
MDA5: – Melanoma Differentiation-Associated Protein 5
MAF: – Minor Allele Frequency
MAS: – Macrophage Activation Syndrome
NF-kB: – Nuclear Factor Kappa-light-chain-enhancer of Activated B cells
PMID: – PubMed Identifier
PRISMA: – Preferred Reporting Items for Systematic Reviews and Meta-Analyses
RNA: – Ribonucleic Acid
RP-ILD: – Rapidly Progressive Interstitial Lung Disease
SJS: – Stevens-Johnson Syndrome
SLE: – Systemic Lupus Erythematosus
SNP: – Single Nucleotide Polymorphism
T1D: – Type 1 Diabetes
T1DM: – Type 1 Diabetes Mellitus
UC: – Ulcerative colitis
VEOIBD: – Very Early Onset Inflammatory Bowel Disease

## 1 Introduction

**Fig. 1:**
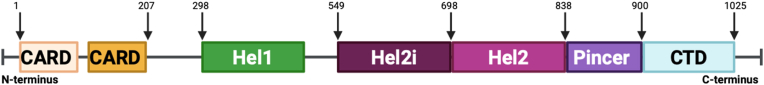
Structure of the Melanoma Differentiation Associated 5 protein (MDA5). MDA5 is composed of 1025 amino acids. The domain regions are delimited with the amino acid coordinates. The N terminus has two tandem caspase recruitment domains (CARD), are responsible for the activation of MAVS. The central region features a Helicase region composed of Hel1 and Hel2, which drive ATP hydrolysis in response to RNA recognition, and an intermediate Hel2i domain that aids in detecting dsRNA (double-strand RNA). The Pincer domain provides structural support and facilitates ATPase activity. The C-terminal domain is involved in dsRNA binding together with the helicase domains [7].

**Fig. 2:**
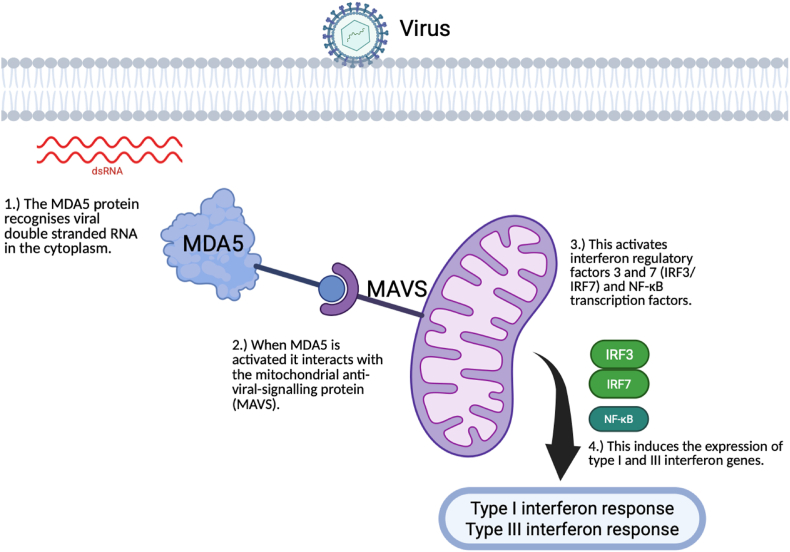
Molecular function of MDA5. MDA5 triggers a type I and III interferon response when it is activated by viral dsRNA. The MDA5 protein recognises viral double stranded RNA in the cytoplasm. When MDA5 is activated, it interacts with the mitochondrial anti-viral signalling protein (MAVS), activating interferon regulatory factors 3 and 7 (IRF3/IRF7) and NF-kB transcription factors. This induces the expression of type I and type III interferon genes, triggering the interferon response [2].

The interferon induced with helicase C domain 1 gene (*IFIH1*) on chromosome 2 [1] encodes for the melanoma differentiation-associated 5 (MDA5) protein [2], an immune receptor involved in the innate immune antivirus responses [3]. MDA5 recognises long double-stranded RNA (dsRNA) from the genome of dsRNA viruses and from replication intermediates of positive sense single-strand RNA (ssRNA) viruses [2,4], triggering inflammatory responses through the induction of type I interferon (IFN) expression [3]. Its protein structure consists of two N-terminal Caspase Activation and Recruitment Domains (CARDs), two Helicase domains (Hel1 and Hel2) separated by an insert domain (Hel2i), then a pincer and a C-terminal domain that is responsible for RNA sensing and binding together with the helicase domain [2,5] (Fig. 1). Upon activation, MDA5 forms polymers [6] and interacts with the Mitochondrial Anti-Viral Signalling protein (MAVS) triggering a signalling cascade that results in the activation of a series of regulatory and transcription factors which subsequently leads to the production of type I IFNs [2] (Fig. 2).

Gain-of-function variants in *IFIH1* have been associated with a range of IFN mediated autoinflammatory diseases, including Aicardi-Goutières syndrome, Singleton Merton syndrome and rare ‘monogenic’ systemic lupus erythematosus (SLE) [2,8,9]. Several studies reported rare gain-of-function mutations in *IFIH1* as a cause of rare ‘monogenic’ SLE as well as disease-associated risk alleles affecting *IFIH1* in more common multifactorial SLE that are not ‘strong’ enough to cause disease independently [10]. Development of these hyperinflammatory diseases have been linked to enhanced or spontaneous activation of MDA5 and, consequently, chronically increased expression of type I IFNs that may induce a vicious cycle of immune activation, tissue inflammation and damage, release of nuclear components through inflammatory cell death, and the production of autoantibodies as a consequence [[11], [12], [13], [14]].

Conversely, loss-of-function mutations in *IFIH1* as well as the presence of anti-MDA5 antibodies have either been associated with increased susceptibility to recurrent and/or severe virus infections [2,[15], [16], [17]]. In seeming contrast, *IFIH1* loss-of-function variants have also been associated with either protection from or increased risk for pathophysiologically complex autoimmune/inflammatory diseases such as type 1 diabetes [18] or inflammatory bowel disease (IBD) [7]. Anti-MDA5 antibody positivity has been linked with increased clinical severity of the systematic inflammatory disease dermatomyositis, including severe pulmonary involvement [19,20]. In this systematic review, the link between *IFIH1* loss-of-function variants, the presence of (likely inactivating) anti-MDA5 antibodies and the development of infectious or autoimmune/inflammatory diseases will be explored.

## 2 Methods

A systematic literature review, following ‘Preferred Reporting Items for Systematic Reviews and Meta-Analyses’ (*PRISMA*) guidance [21], was conducted in March 2025 accessing the *PubMed* and *Medline (Ovid)* databases (Supplement Tables 1 and 2**)**. The search terms used were [*IFIH1* OR MDA5 OR MDA-5 AND loss of function mutations OR loss-of-function mutations]. Studies included in this review explored the role of loss-of-function mutations in *IFIH1*/MDA5 in the innate immune response. Studies that discussed how anti-MDA5 antibodies contribute to the development of autoimmune diseases were also included. Individual case reports were included as the findings were deemed important in understanding the involvement of *IFIH1*/MDA5 in immune regulation. Additional sources were used to provide a deeper understanding on the topic, which were then included in the introduction and discussion sections of the review, as well as the reference list.

The genome databases *Ensembl* [22] and *gnomAD v4.1* [23] were accessed to consistently name *IFIH1* variants and to identify minor allele frequencies (MAF) across ancestries.

Exclusion criteria for this review included manuscripts that were not published in English language, narrative or systematic reviews, reports and studies focussing on zoonoses, and manuscripts that discussed other proteins involved in the innate immune response, without inclusion of MDA5 (for example *ADAR1*). Additionally, reports focussing on gain-of-function variants in *IFIH1*, without discussion of loss-of-function mutations, were excluded. An additional systematic search was conducted in January 2026 [anti-MDA5 OR anti-MDA5 antibodies AND COVID-19 OR virus OR influenza] **(**Supplement Table 3**)** to capture work investigating the link between anti-MDA5 antibodies in the context of SARS-CoV2 or influenza infections (namely, their impact on disease courses, and post-infectious anti-MDA5 positivity with associated autoimmune phenomena).

All records were imported to *Endnote* (Endnote 21 Clarivate, USA) and duplicates were removed. First, titles of studies were assessed to determine whether they were relevant to the focus of the review. Then, abstracts were assessed for relevance with a focus on whether they included details related to loss-of-function mutations in *IFIH1* and MDA5, as well as the context of infection, the autoimmune or inflammatory response. Following the abstract review, full texts and supplementary material for the remaining studies were analysed. All manuscripts were first screened by II, full-text review was delivered by II and CMH. The full text review involved assessing the type of study (e.g. observational or experimental) and determining whether it was conducted in human or mouse models.

## 3 Results and discussion

**Fig. 3:**
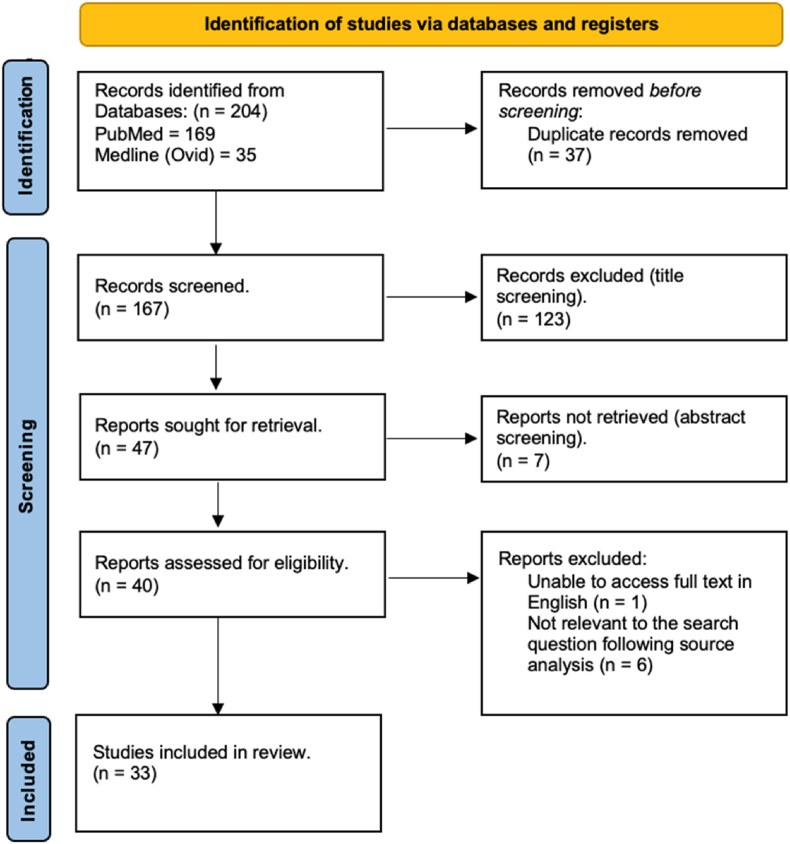
PRISMA diagram. Of the 33 reports included in this systematic review, 13 referred to specific loss-of-function mutations in the *IFIH1* gene [2,3,12,18,7,[24], [25], [26], [27], [28],[29], [30], [31],^32^] discussed the general effect of loss-of-function *IFIH1* mutations on the immune system or the inflammatory response [33,[34], [35], [36],[37], [38], [39], [40],[41], [42], [43]], and 9 manuscripts discussed inactivating anti-MDA5 antibodies [19,20,[44], [45], [46],^47^,[48], [49], [50]]. Overall, 16 individual *IFIH1* loss-of-function mutations were reported across the literature (Table 1, Tab 5 - supplement).

**Fig. 4:**
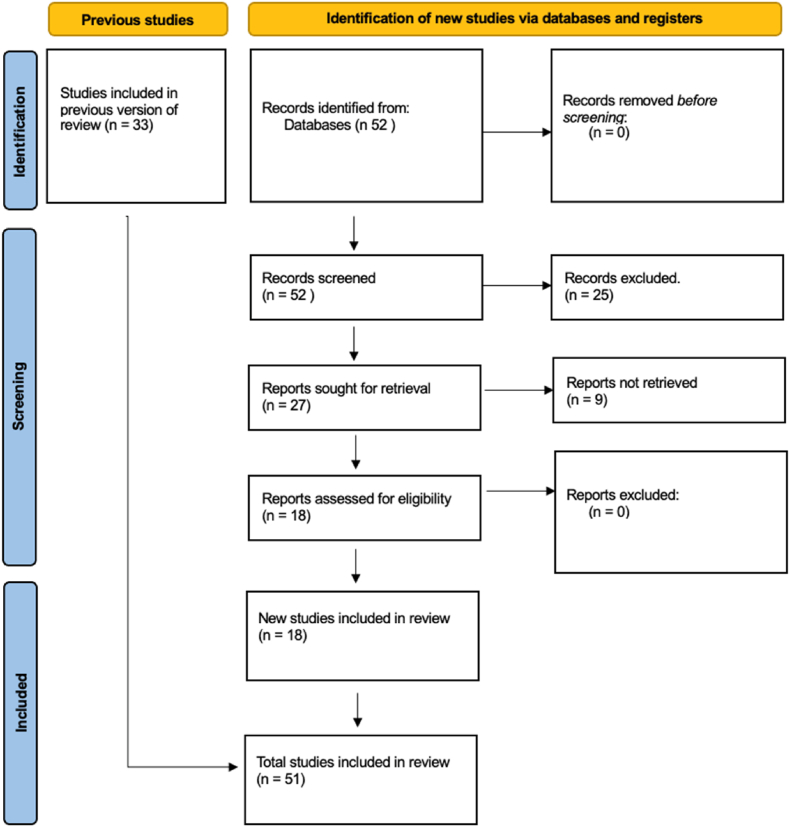
PRISMA diagram (second search). An additional search was conducted to capture studies investigating anti-MDA5 antibody positivity in COVID-19. The same exclusion criteria were applied that were used for the original search. Of the 18 additional studies identified, 11 discussed anti-MDA5 antibodies post SARS-CoV2 infection [[15], [16], [51], [52], [53], [54], [55], [56], [57], [58], [59]] discussed anti-MDA5 positive dermatomyositis following SARS-CoV2 vaccination [60,[61], [62], [63], [64], [65], [66]].

In total, 204 manuscripts were identified, 169 manuscripts from *PubMed* and 35 from *Medline* (Ovid) searches. Following removal of duplicates using *Endnote*, titles of 167 records were screened, reducing the number of relevant sources to 123 for abstract and 47 for full text review. During this stage, an additional 14 manuscripts were excluded because they did not discuss loss-of-function variants affecting *IFIH1*, leaving 33 sources to be included in the systematic review (Fig. 3, Supplement Table 4). A second search was conducted on *PubMed* identifying 52 records, after screening a total of 18 records have been added, resulting in a total of 51 studies being included in the review **(**Supplement Table 3, Fig. 4).

**Fig. 5:**
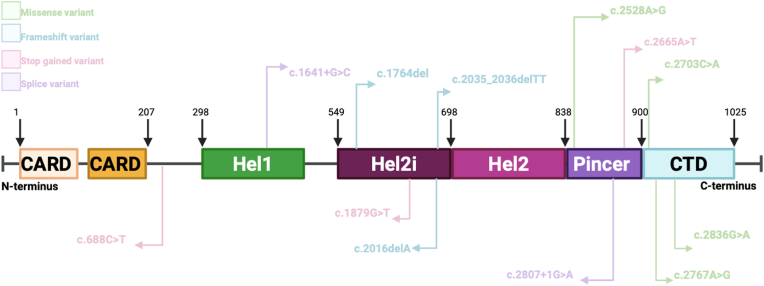
Protein domains and effects of *IFIH1* loss-of-function variants. The MDA5 protein contains two N-terminal caspase recruitment domains (CARD), a central helicase region (Hel1, Hel2 and the intermediate Hel2i domain) and the Pincer and C-terminal domains (CTD) at the C terminus [7]. MDA5 is composed of 1025 amino acids. The domain regions are marked with the amino acid coordinates. In this figure, 12 of the identified loss-of-function variants have been annotated, showing their location on the MDA5 protein [18]. This diagram was inspired by a diagram from Downes K et al. Reduced expression of *IFIH1* is protective for type 1 diabetes and data from Ensembl was used to add additional variants.

A total of 16 loss-of-function variants affecting *IFIH1* have been identified and associated with differential impact on protein function and/or disease association (Table 1, Table 2, Fig. 5, Supplement Table 4).

**Table 1:** *IFIH1* loss-of-function variants.

c.DNA	Protein	rsID	Global MAF	Functional impact	Associated diseases	
					Protective Effect	Increased susceptibility
c.2016delA	p.Asp673∗	rs773033563	**0.00019**	**Frameshift variant.**		**IBD** [2]**,** MIS-C [2], VEOIBD [7]
c.1641+1G > C	**IVS8+1**	rs35337543	0.00003677	Splice donor.	T1DM association [18,24]	severe COVID-19, MIS-C, viral infections [25], [2,26,27]
c.2807+1G > A	p.Ile872Ter **IVS14 + 1**	rs35732034	**0.009**	**Splice donor.**	**hypothyroidism, T1DM** [18,24,28]	**Severe COVID-19 (2), VEOIBD** [7]**, viral infections** [26,33]
c.2665A > T	p.Lys889Ter	rs1252022173	**0.000001369**	**Stop gained.**		**Increased susceptibility to infections.** [26,29]
c.1879G > T	p.Glu627Ter	rs35744605	**0.005**	**Stop gained.**	**hypothyroidism, T1DM, IBD** [3,18,24,28,30]	**VEOIBD, viral infections** [7,26,33,29]
c.2836G > A	p.Ala946Thr	rs1990760	0.431	Missense variant.	Hypothyroidism, coronary artery disease, T1DM, autoimmunity [18,28,30],	chronic viral infections [31] COVID-19 (34)
c.2767A > G	p.Ile923Val	rs35667974	0.017	Missense variant.	IBD, protects from T1DM [3,24,33,30]	
c.2703C > A	p.Ile901Asn		**-**	**Missense** variant.		**Severe COVID-19** [2]
c.1764del	p.Ala589∗	rs553669430	**0.000391**	**Frameshift variant.**	[27]	
c.769+3A > G	p.Leu257∗		**-**	**Splice variant.**	[27]	
c.2035_2036delTT	p.Val679∗		**-**	**Frameshift variant.**		**VEOIBD** [7]
c.688C > T	p.Gln230Ter	rs771251917	**0.00004858**	**Stop gained.**		**VEOIBD** [7]
c.2044+2T > C		rs201026962	**0.0001469**	**Splice donor.**	Hypothyroidism [28]	
c.454-1G > T		rs148590996	0.00004524	Splice acceptor.	Hypothyroidism [28]	
c.2528A > G	p.His843Arg	rs3747517	0.298	Missense variant.	[18]	
c.770-4185C.T		rs13023380	0.000006589	Intron variant.	[18]	

The database Ensembl was used to collect the data. MAF: minor allele frequency; N/A: not available: Refs: references; IBD: Inflammatory bowel disease; MIS-C: Multisystem inflammatory syndrome in children; VEO-IBD: Very Early Onset Inflammatory Bowel Disease; T1DM: Type 1 diabetes; UC: Ulcerative Colitis. Variants without a reported minor allele frequency do not have a rs number, which accounts for the missing data.

**Table 2:** Minor Allele Frequencies (MAFs) of *IFIH1* variants.

c.DNA name	rsDNA	Reference Allele	Altered Allele	AFR	AMR	NFE	MID	EAS	ALL
c.2016delA	rs773033563	A	del	0.00003	0.00023	0.00005	0.002	0.00003	0.00019
c.1641+1G > C	rs35337543	G	C	0.002 (G)	0.01(G)	0.000002942 (T)	0.013 (G)	0.00002552 (G)	0.00003677 (T)
c.2807+1G > A	rs35732034	G	A	0.001 (T)	0.003 (T)	0.009 (T)	0.007 (T)	0.021 (T)	0.009 (T)
c.2665A > T	rs1252022173	A	T	0	0.0000224 (A)	0.0000009 (A)	0	0	0.000001369 (A)
c.1879G > T	rs35744605	G	T	0.001 (A)	0.001 (A)	0.007 (A)	0.001737 (A)	0.00002522 (A)	0.005 (A)
c.2836G > A	rs1990760	G	A	0.166 (T)	0.426 (T)	0.391 (C)	0.498 (C)	0.202 (T)	0.431 (C)
c.2767A > G	rs35667974	A	G	0.002 (C)	0.002 (C)	0.02 (C)	0.0003489 (C)	0.00002528 (C)	0.017 (C)
c.2703C > A	-	C	A	-	-	-	-	-	-
c.1764del	rs553669430 (minor allele in all populations is the longer repeat TTTTTTTTT)	Reference repeat	Longer T repeat	0.006	0.0002644	0.000002005	0.000002002	0.002	0.000391
c.769+3A > G	-	A	G	-	-	-	-	-	-
c.2035_2036delTT	-	TT	del	-	-	-	-	-	-
c.688C > T	rs771251917	C	T	0	0	0.00006296 (A)	0	0	0.00004858 (A)
c.2044+2T > C	rs201026962	T	C	0	0	0.0001895 (G)	0	0	0.0001469 (G)
c.454-1G > T	rs148590996	G	T	0	0	0.00005674 (A)	0	0	0.00004524 (A)
c.2528A > G	rs3747517	A	G	0.39 (T)	0.187 (T)	0.275 (T)	0.306 (T)	0.344 (C)	0.298 (T)
c.770-4185C.T	rs13023380	C	T	0.097 (A)	0.361 (A)	0.00001472 (T)	0.459 (A)	0.008 (A)	0.000006589 (T)

Minor Allele Frequencies (MAFs) as reported in gnomAD v4.1 (exomes or genomes) and accessed via Ensembl. Variant frequencies were derived from gnomAD exomes v4.1 [11] and v4.1 [12]. MAFs are reported for individual ancestries AFR (African), AMR (Latino), NFE (Non-Finnish European), MID (Middle Eastern), EAS (East Asian) and ALL (global cohort).

### 3.1 *IFIH1* loss-of-function variants and increased susceptibility to virus infections

***Viral respiratory infections -*** Loss-of-function variants in *IFIH1* have been linked with increased susceptibility to virus infections and enhanced disease severity [2] (Table 1, Table 2, Fig. 5, Supplement Table 4). One study investigated children requiring intensive care support due to respiratory virus infections (namely, with respiratory syncytial virus and human rhinovirus), suggesting that *IFIH1* loss-of-function may result in primary immunodeficiency [26]. Whole exome and RNA sequencing identified three rare *IFIH1* variants associated with severe viral disease and confirmed their functional impact, namely c.2807+1G > A (a splice donor mutation at exon 14 disrupting the 2-bp donor splice site at the 5’ end on the intron likely causing exon skipping or truncation) (MAF: 0.009); c.1879G > T (p.Glu627∗) (MAF: 0.5) and c.1641+1G > C (a frameshift mutation causing partial loss of the dsRNA virus binding helicase domain) (MAF: 0.00004) [26].

Another case report identified a homozygous nonsense mutation IFIH1 c.2665A > T (p.Lys889∗; identified through whole-exome sequencing) that may result in unstable mRNA transcripts or a truncated protein lacking the C-terminal domain (MAF: 0.000001) [29]. This variant associated with recurrent and prolonged virus infections (including EBV) in the reported patient [29].

***SARS-CoV2 –*** Severe acute respiratory syndrome coronavirus 2 (SARS-CoV2) is the pathogen responsible for the Coronavirus Disease 2019 (*COVID-19) pandemic* [67]*.* As for other respiratory RNA viruses (above), loss-of-function mutations in *IFIH1* increase the susceptibility to SARS-CoV2 [2] (Table 1, Table 2, Fig. 5, Supplement Table 4). Several *IFIH1* variants associate with severe infections, including the heterozygous c.1641+1G > C variant (previously reported in association with other respiratory virus infections), the heterozygous frameshift variant c.2016delA (p.D673Ifs∗5; causing premature truncation), and the homozygous c.2807+1G > A splice donor variant (causing exon skipping or truncation) that also presented with the most severe symptoms [2]. Notably, the homozygous c.2807+1G > A (p.His843Arg) variant present in one patient also associated with variable immunodeficiency with glomerulonephritis and bacterial septicaemia [2].

***Human immunodeficiency virus (HIV) –*** HIV is a human pathogenic RNA retrovirus causing the acquired immunodeficiency disease (AIDS) [34]. The cytoplasmic MDA5 immune receptor is involved in the detection of HIV-1 [34]. Genetic variants in *IFIH1* associate with differential susceptibility to HIV infections and disease outcomes, e.g. through chronic inflammation and persistent HIV replication that is due to disruption of the CARD domain responsible for the activation of the type I interferon responses and the associated induction of antiviral effects [34].

***Enterovirus infections –*** *Enterovirus* is a genus of small, positive sense ssRNA viruses associated with several human and mammalian diseases [25], including myocarditis/rhombencephalitis and virus induced type 1 diabetes [35,36]. Serologic studies have distinguished >70 human pathogenic enterovirus serotypes [68]. While most enterovirus infections present with mild manifestations or remain asymptomatic, a small proportion develop severe complications such as myocarditis, myelitis and encephalitis [25]. In such cases, enteroviruses can cross the blood brain barrier and result in meningitis or encephalitis which typically presents as rhombencephalitis [25], an inflammatory disease affecting the brainstem and cerebellum. The aforementioned *IFIH1* c.1641+1G > C (p.L509_E547 del [△exon8]) variant, that has been associated with increased susceptibility and/or adverse disease outcomes in other virus infections, has been reported in a child with severe *enterovirus* rhombencephalitis [25]. Moreover, MDA5-deficiency in mice increases their susceptibility to viral *Enterovirus* infections with *Coxsackievirus B3* (CVB3) [35], uncontrolled virus replication early after infection, enhanced inflammation and tissue damage, subsequently resulting in organ damage to the liver and pancreas [35].

Overall, these studies have demonstrated that impaired MDA5 function leads to the disruption of the innate immune response, predisposing patients to severe and recurrent viral infections.

### 3.2 *IFIH1* loss-of-function and the associated risk for autoimmune/inflammatory diseases

Altered expression of type 1 IFNs has been associated with the development of several autoimmune/inflammatory diseases, including thyroid disease, type 1 diabetes, inflammatory bowel disease (IBD), dermatomyositis (DM), and others [17,18,28,32]. The increased expression of IFN in the context of virus infections may therefore be a link between genetic predisposition of an individual for the development of certain inflammatory diseases and environmental impact [28]. Thus, *IFIH1* loss-of-function variants are interesting candidates in the search for genetic factors altering an individual's risk for the development of post-viral autoimmune phenomena.

The loss-of-function splice variant in *IFIH1* c.1641+G > C (rs35337543), that has been associated with increased susceptibility to RNA virus infections and severe disease courses, may also protect from autoimmune thyroid disease, type 1 diabetes, psoriasis and vitiligo (UK biobank) [28]. The same study suggested that the common *IFIH1* missense variant c.2719G > A may be associated with a lower risk of coronary heart disease [28], suggesting that reduced IFN expression associated with *IFIH1* loss-of-function may alter the risk for a range of autoimmune/inflammatory diseases.

Conversely, one study identified loss-of-function *IFIH1* variants in three boys with periodic fever and autoinflammatory disease from the Middle East [27]. Mutations identified were heterozygous *IFIH1* loss-of-function variants c.1641+1G > C (a frameshift mutation previously associated with severe virus infections [26]) (MAF:0.00004), c.1764del (p.Ala589Leufs∗16; a frameshift mutation causing a premature stop codon) (MAF 0.0004) and c.769+3A > G (splice variant that disrupts normal splicing), highlighting that heterozygous *IFIH1* loss-of-function variants may also contribute to autoinflammatory disease phenotypes [27].

***Type 1 diabetes –*** Type 1 diabetes (T1D) is a T cell-mediated autoimmune/inflammatory disease that results in the destruction of pancreatic β cells. Its pathophysiology is complex and involves several environmental, genetic, and immune-mediated factors [30], including upregulation of IFN responses in the context of virus infections [37].

This systematic review identified 10 reports discussing *IFIH1* loss-of-function variants as a ‘protective’ factor in T1D, but mechanisms associated with the protective role of MDA5 are complex and incompletely understood. The cytoplasmic MDA5 receptor is expressed in pancreatic islet cells and is upregulated by cytoplasmic RNA [38]. When MDA5 interacts with RNA viruses (such as coxsackievirus B), it causes local inflammation in the pancreas, resulting in a loss of β cells and, in some cases, T1D [39]. In mice, MDA5 expression associates with pronounced regulatory T cell responses (rather than the ‘usual’ effector T cell response) protecting them from the development of immune-mediated diabetes following RNA virus exposure [36]. Moreover, reduced MDA5 expression associates with decreased chemokine production from pancreatic β-cells, reduced islet inflammation and diabetes [38].

In line with these reports, several studies in humans suggest that reduced MDA5 expression/function delays or protects from the development of T1D. Both *IFIH1* c.1879G > T (p.Glu627∗) and c.2767A > G (p.Ile923Val) affect splicing and protect from T1D [30]. The c.1879G > T (p.Glu627∗) variant results in deletion of the MDA5 C-terminus and its dsRNA binding capacity. The c.2767A > G mutation affects the C-terminal domain by reducing its catalytic activity (but not its ability to bind dsRNA *per se*) [3]. Both *IFIH1* variants reduce the functional activity of MDA5, subsequent IFN expression and the associated risk of developing type 1 diabetes [30]. Moreover, the rare splice variants c.1641+1G > C and c.2807+1G > A associate with reduced diabetes risk [18].

Taken together, these diabetes-associated *IFIH1* variants mediate increased MDA5 activity, contributing to increased numbers of human monocytes–derived dendritic cells, effector CD4^+^ T cells [24], and the generation of autoantibodies targeting pancreatic β cells [38]. Notably, in fulminant T1D with rapid β cell destruction, MDA5 in the islet cells is enhanced, further suggesting its central involvement in the pathomechanism [40]. However, the situation may be more complex as the common c.2719G > A (rs1990760) variant, resulting in p.A946T substitution, reduced MDA5 receptor expression and reduced type III IFN response to coxsackievirus B3 [31], associates with increased diabetes risk [18]. This suggests that tight regulation of MDA5 expression/function is necessary to sustain immune balance and pancreas integrity.

***Inflammatory bowel disease –*** The association between loss-of-function variants in *IFIH1* and the development of inflammatory bowel disease (IBD) was explored by two studies included here. Defects in the innate immune system, due to genetic variants that impair the sensing of bacteria and/or viruses, may be associated with the development of IBD [7]. One study identified five individual rare *IFIH1* loss-of-function variants in eight patients with very early-onset IBD (before 6 years) [7]. The IBD-associated homozygous *IFIH1* c.2016delA (p.Asp673∗) frameshift variant (MAF: 0.0002) causes complete MDA5 deficiency, whereas heterozygous *IFIH1* c.2035_2036delTT (p.Val679∗), c.2807+1G > A (p.Ile872Ter) (MAF: 0.009), c.688C > T (p.Gln230Phe) (MAF: 0.005) and c.1879G > T (p.Glu627∗) (MAF: 0.005) associate with partial MDA5 deficiency [7]. Complete MDA5 deficiency causes life-threatening neonatal-onset pan-gastrointestinal immune-mediated-enteropathy alongside recurrent bacterial and viral infections. In children experiencing partial MDA5 deficiency, a milder IBD phenotype was observed that also presented after the neonatal period in the absence of recurrent and/or severe infections [7]. Another study investigated three IBD-associated *IFIH1* loss-of-function variants [32]: c.1879G > T (p.Glu627∗), c.2767A > G (altering a highly conserved amino acid, p.Ile923Val) and the previously discussed c.2807+1G > A (p.Ile872Ter) variant (affecting an essential splicing position) [32]. The study demonstrated that loss of MDA5 function impacts on intestinal health, as orchestrated innate immune responses to enteric viruses (induced by functional MDA5) are necessary for intestinal epithelial cell function and integrity [32].

Taken together, studies suggest that impaired viral sensing may contribute to the pathogenesis of IBD and innate immune dysregulation, resulting in increased susceptibility to very early-onset IBD and, in cases of complete MDA5 deficiency, bacterial and/or viral infections [7].

### 3.3 ‘Loss-of-function’ through anti-MDA5 antibodies

***Dermatomyositis and anti-MDA5 antibodies --*** Dermatomyositis (DM) is a multisystem autoimmune/inflammatory disease characterised by myositis and skin inflammation that involve disease-specific pathological antigen responses [20]. In a subset of patients, MDA5 has been identified as a DM-associated autoantigen that associates with vasculopathy. Patients with anti-MDA5 antibodies are more likely to have ‘mild’ muscle disease but are at greater risk for rapidly progressive interstitial lung disease (ILD) and/or other complications [19,20]. Among adult-onset DM patients, anti-MDA5 antibody seropositivity associates with a less favourable prognosis when compared to seronegative disease [[44], [45], [46]]. Three different subgroups of anti-MDA5 positive DM have been described, namely, rapidly progressing (RP) ILD, ‘rheumatic’ DM and vasculopathic DM [69]. The RP-ILD subgroup is associated with poor prognosis secondary to severe lung involvement, early and high mortality. Anti-MDA5 positive ‘rheumatic’ DM is characterized by dermatological and joint involvement that has good prognosis. Lastly, vasculopathic DM corresponds to patients with severe skin vasculopathy, including skin ulcers, digital necrosis and calcinosis, with some patients developing RP-ILD. Thus, vasculopathic DM carries an ‘intermediate’ prognosis between other subtypes of MDA5 antibody positive DM [69].

Notably, anti-MDA5 antibodies are also present in approximately 7-32% of patients with juvenile-onset DM (JDM), with higher frequencies noted in Asian populations compared to UK or North American cohorts [47,70]. While data are slightly more limited, as in adults, the presence of anti-MDA5 antibodies associates with ILD and less severe muscle involvement [47,70].

In the absence of functional laboratory studies, the exact contribution of anti-MDA5 antibodies to the disease mechanism remains somewhat unclear but may be related to delayed or incomplete virus clearance. In response to the detection of cytosolic dsRNA, MDA5 induces the transcription of type I IFNs. MDA5 itself is inducible by IFNs and therefore sits within a positive feedback loop promoting pathogen clearance through inflammation [17]. Increased expression of MDA5 in a subset of patients with virus infections may result in loss of its subcellular localization of MDA5, tissue damage and a break of immune tolerance (including the production of anti-MDA5 antibodies). Delayed IFN production may impair virus clearance and (which may appear paradoxical) subsequently result in increased inflammation, tissue damage (including ILD), and vasculopathy [17]. This possible link between virus susceptibility and DM, including phenotypic variability, may also contribute to the abovementioned genetically determined susceptibility to coxsackie virus infections. Coxsackie B viruses have been linked with the development of DM in the late 1980 [71]. The functional link of (genetically determined or autoantibody-mediated) reduced IFN expression and subsequently enhanced DM expression and/or severity remains hypothetical as functional studies are lacking, and no information exists on anti-MDA5 autoantibody positivity in coxsackie-associated DM [72].

Notably, immune dysregulation and hyperinflammation similar to that seen in DM have been reported in patients with severe COVID-19 and ILD. Indeed, DM and COVID-19 share systemic symptoms, blood cytokine profiles and radiographic presentations on chest X-rays and/or CT scans reflecting ILD [73]. The IFN-mediated hyperinflammatory response triggered by COVID-19 overlaps with anti-MDA5 positive DM, and both diseases respond to similar immunosuppressive treatment regimens. Moreover, preliminary evidence of molecular mimicry and increased flare rated of DM patients during the COVID-19 pandemic further support a potential pathogenic connection [73]. Another study focussing on the NF-kB pathway demonstrated that loss-of-function variants impair activation of the transcription factor RelB, leading to reduced expression of the Autoimmune Regulator (AIRE) in medullary thymic epithelial cells. AIRE mediates the presentation of organ-specific self-proteins to lymphocytes during their differentiation and priming. Thus, reduced AIRE expression allows autoreactive T cells to escape, disrupting T cell tolerance and resulting in the production of autoantibodies against, e.g., type I interferons [74]. This suggests that loss-of-function mutations in immune regulatory pathways that impair central T cell tolerance may predispose also to the production of anti-MDA5 antibodies, rather than these antibodies arising solely from secondary inflammation. However, the exact underlying pathology explaining why some patients exhibit these autoantibodies and how they contribute to distinct and severe clinical phenotypes remains unknown.

***Interrelations between virus exposure and anti-MDA5 antibodies --*** Increasing evidence indicates an association between SARS-CoV2 exposure, through infection or vaccination, and the development of anti-MDA5 antibodies [16,51]. As previously discussed, anti-MDA5 positive dermatomyositis and COVID-19 share clinical, pathogenic, and radiological features which led to the proposal of COVID-19 as a ‘human model’ of anti-MDA5 positive autoimmune disease [75,76]. Following SARS-CoV2 exposure, MDA5 activation results in innate immune activation and type I and type III IFN responses [16,51]. Notably, excessive activation of type I IFNs is implicated in the pathogenesis of several autoimmune diseases, including anti-MDA5 positive DM [60].

Anti-MDA5 antibodies are detectable in the serum of some patients during the acute phase and after COVID resolution, and higher titres correlate with clinical severity and reduced survival [77]. Several case reports described anti-MDA5 positive DM arising after SARS-CoV2 infections [51,[52], [53], [54], [55], [56]]. Environmental triggers, such as SARS-CoV2 exposure, have been suggested as contributors to anti-MDA5 positive DM in genetically susceptible people [56]. This is supported by the seasonal variation of anti-MDA5 positive DM with peak incidences during the winter months when respiratory virus infections also peak [51]. The presence of anti-MDA5 antibodies may not only distinguish patients with severe COVID-19 from milder infections, they also associate with treatment response and disease outcomes in patients with DM and progressive ILD [16]. Similarly, the presence of anti-MDA5 antibodies may identify a subset of patients at risk for severe pulmonary involvement during SARS-CoV2 infection [77,56,78]. A study conducted in China reported that the SARS-CoV2 infection rates (Omicron variant) were higher in patients with anti-MDA5 positive DM when compared to the general population, suggesting impaired anti-viral responses through MDA5 inhibition [15]. However, while patients with anti-MDA5 positive DM were more vulnerable to SARS-CoV2 infections, clinical symptoms of COVID-19 did not differ when compared to people without anti-MDA5 antibodies. This may have been due to the relatively low virulence of the Omicron variant or because most patients with DM in the study were in an inactive state before contracting SARS-CoV2 [15].

Lastly, there have been several case reports of anti-MDA5 positive DM in previously healthy individuals following SARS-CoV2 vaccination (when specified, mRNA vaccines were used in most studies; one manuscript used an inactivated SARS-CoV2 vaccine) [[61], [62], [63], [64], [65], [66]]. In addition to possible associations between SARS-CoV2 vaccinations and anti-MDA5 positive DM, one study suggested anti-MDA5 positive DM patients not to receive sufficient protective effect from vaccination [15]. As functional studies are lacking, it remains speculation whether this was the result of excessive IFN production resulting in disease expression in people genetically predisposed to develop DM.

In conclusion, SARS-CoV2 exposure associates with the induction of anti-MDA5 antibodies in some people which is linked to more severe COVID-19 phenotypes, progressive ILD, increased mortality, and anti-MDA5 mediated autoimmunity [56].

## 4 Conclusions

The innate immune receptor MDA5 (encoded by the *IFIH1* gene) plays a central role in host defence against RNA viruses and immune homeostasis. Loss-of-function mutations in *IFIH1* have been associated with higher infectious disease susceptibility and risk for complications, but also with protection from some and increased risk for other autoimmune/inflammatory diseases. Furthermore, functional deletion of MDA5 through autoantibodies affects the phenotype of inflammatory myopathies, potentially through pathogen-mediated mechanisms. Observations underscore the importance of a tightly regulated inflammatory response mediated by MDA5 (*IFIH1*) for immune homeostasis and the prevention of tissue damage. Additional experimental work is necessary to understand the contribution of genetic variation at *IFIH1* to infectious and autoimmune/inflammatory diseases, which may result in risk stratification tools and new treatment options.

## CRediT authorship contribution statement

**Isabelle Ince:** Writing – review & editing, Writing – original draft, Visualization, Project administration, Methodology, Investigation, Formal analysis, Data curation, Conceptualization. **Francesca Sposito:** Writing – review & editing, Writing – original draft, Validation, Project administration, Investigation. **Amandine Charras:** Writing – review & editing, Writing – original draft, Supervision, Formal analysis, Conceptualization. **Liza J. McCann:** Writing – review & editing, Writing – original draft, Supervision, Formal analysis, Data curation. **Christian M. Hedrich:** Writing – review & editing, Writing – original draft, Supervision, Resources, Project administration, Methodology, Investigation, Formal analysis, Data curation, Conceptualization.

## Declaration of generative AI use

No AI tools have been used to generate this manuscript.

## Funding sources

CMH is supported by Arthritis UK, LUPUS UK, SRUK, the Alder Hey Children's Charity, Funding Autoimmune Research (FAIR), the Parry Family Charitable Foundation, the NIHR GOSH Biomedical Research Centre, the NIHR Alder Hey Children's Clinical Research Facility, and Merck (MISP).

## Declaration of competing interest

The authors declare the following financial interests/personal relationships which may be considered as potential competing interests: CMH receives unrestricted research support from Merck (MISP) to study kidney inflammation in SLE. Authors declare no conflict of interest in relation to the work presented.

## Data Availability

Data will be made available on request.
