# Supplementary material for: How loss-of-function mutations in IFIH1 contribute to infectious and/or inflammatory disease – a systematic review
Source: J Transl Autoimmun. 2026 Feb 4;12:100353. doi: 10.1016/j.jtauto.2026.100353 (PMC12906142; doi:10.1016/j.jtauto.2026.100353)
Supplement: Multimedia component 1 [file mmc1.docx]

**Supplementary material**

**Supplement Table 1: Search Strategy PubMed** (09/03/2025).

| #1 | **IFIH1**  1776 results | "IFIH1"[All Fields] |
| --- | --- | --- |
| #2 | **MDA5 OR MDA-5**  2987 results | "MDA5"[All Fields] OR "MDA-5"[All Fields] |
| #3 | **Loss of function OR Loss-of-function**  760,598 results | ("Loss"[All Fields] AND ("functional"[All Fields] OR "functional s"[All Fields] OR "functionalities"[All Fields] OR "functionality"[All Fields] OR "functionalization"[All Fields] OR "functionalizations"[All Fields] OR "functionalize"[All Fields] OR "functionalized"[All Fields] OR "functionalizes"[All Fields] OR "functionalizing"[All Fields] OR "functionally"[All Fields] OR "functionals"[All Fields] OR "functioned"[All Fields] OR "functioning"[All Fields] OR "functionings"[All Fields] OR "functions"[All Fields] OR "physiology"[MeSH Subheading] OR "physiology"[All Fields] OR "function"[All Fields] OR "physiology"[MeSH Terms])) OR "Loss-of-function"[All Fields] |
| #4 | **Autoimmune**  279,576 results | "autoimmune"[All Fields] OR "autoimmunity"[MeSH Terms] OR "autoimmunity"[All Fields] OR "autoimmunities"[All Fields] OR "autoimmunization"[All Fields] OR "autoimmunizing"[All Fields]  **Translations**  **Autoimmune:** "autoimmune"[All Fields] OR "autoimmunity"[MeSH Terms] OR "autoimmunity"[All Fields] OR "autoimmunities"[All Fields] OR "autoimmunization"[All Fields] OR "autoimmunizing"[All Fields] |
| #5 | **Inflammatory response**  362,711 results | ("inflammatories"[All Fields] OR "inflammatory"[All Fields]) AND ("response"[All Fields] OR "responses"[All Fields] OR "responsive"[All Fields] OR "responsiveness"[All Fields] OR "responsivenesses"[All Fields] OR "responsives"[All Fields] OR "responsivities"[All Fields] OR "responsivity"[All Fields])  **Translations**  **Inflammatory:** "inflammatories"[All Fields] OR "inflammatory"[All Fields]  **response:** "response"[All Fields] OR "responses"[All Fields] OR "responsive"[All Fields] OR "responsiveness"[All Fields] OR "responsivenesses"[All Fields] OR "responsives"[All Fields] OR "responsivities"[All Fields] OR "responsivity"[All Fields] |
| #6 | **SLE**  44,833 results | "SLE"[All Fields] |
| #7 | **#1 OR #2**  (IFIH1 OR MDA5 OR MDA-5)  3,547 results | "IFIH1"[All Fields] OR "MDA5"[All Fields] OR "MDA-5"[All Fields] |
| #8 | **#3 AND #7**  (IFIH1 OR MDA5 OR MDA-5) AND (Loss of function OR Loss-of-function)  169 results | (("Loss"[All Fields] AND ("functional"[All Fields] OR "functional s"[All Fields] OR "functionalities"[All Fields] OR "functionality"[All Fields] OR "functionalization"[All Fields] OR "functionalizations"[All Fields] OR "functionalize"[All Fields] OR "functionalized"[All Fields] OR "functionalizes"[All Fields] OR "functionalizing"[All Fields] OR "functionally"[All Fields] OR "functionals"[All Fields] OR "functioned"[All Fields] OR "functioning"[All Fields] OR "functionings"[All Fields] OR "functions"[All Fields] OR "physiology"[MeSH Subheading] OR "physiology"[All Fields] OR "function"[All Fields] OR "physiology"[MeSH Terms])) OR "Loss-of-function"[All Fields]) AND ("IFIH1"[All Fields] OR ("MDA5"[All Fields] OR "MDA-5"[All Fields]))  **Translations**  **function:** "functional"[All Fields] OR "functional's"[All Fields] OR "functionalities"[All Fields] OR "functionality"[All Fields] OR "functionalization"[All Fields] OR "functionalizations"[All Fields] OR "functionalize"[All Fields] OR "functionalized"[All Fields] OR "functionalizes"[All Fields] OR "functionalizing"[All Fields] OR "functionally"[All Fields] OR "functionals"[All Fields] OR "functioned"[All Fields] OR "functioning"[All Fields] OR "functionings"[All Fields] OR "functions"[All Fields] OR "physiology"[Subheading] OR "physiology"[All Fields] OR "function"[All Fields] OR "physiology"[MeSH Terms]  **Warnings**  (Loss **of** function OR Loss-of-function) AND ((IFIH1) OR (MDA5 OR MDA-5))  **Stop word:** of |
| #9 | **#4 AND #8**  (IFIH1 OR MDA5 OR MDA-5) AND (Loss of function OR Loss-of-function) AND (autoimmune)  45 results | ("autoimmune"[All Fields] OR "autoimmunity"[MeSH Terms] OR "autoimmunity"[All Fields] OR "autoimmunities"[All Fields] OR "autoimmunization"[All Fields] OR "autoimmunizing"[All Fields]) AND ((("Loss"[All Fields] AND ("functional"[All Fields] OR "functional s"[All Fields] OR "functionalities"[All Fields] OR "functionality"[All Fields] OR "functionalization"[All Fields] OR "functionalizations"[All Fields] OR "functionalize"[All Fields] OR "functionalized"[All Fields] OR "functionalizes"[All Fields] OR "functionalizing"[All Fields] OR "functionally"[All Fields] OR "functionals"[All Fields] OR "functioned"[All Fields] OR "functioning"[All Fields] OR "functionings"[All Fields] OR "functions"[All Fields] OR "physiology"[MeSH Subheading] OR "physiology"[All Fields] OR "function"[All Fields] OR "physiology"[MeSH Terms])) OR "Loss-of-function"[All Fields]) AND ("IFIH1"[All Fields] OR ("MDA5"[All Fields] OR "MDA-5"[All Fields])))  **Translations**  **Autoimmune:** "autoimmune"[All Fields] OR "autoimmunity"[MeSH Terms] OR "autoimmunity"[All Fields] OR "autoimmunities"[All Fields] OR "autoimmunization"[All Fields] OR "autoimmunizing"[All Fields]  **function:** "functional"[All Fields] OR "functional's"[All Fields] OR "functionalities"[All Fields] OR "functionality"[All Fields] OR "functionalization"[All Fields] OR "functionalizations"[All Fields] OR "functionalize"[All Fields] OR "functionalized"[All Fields] OR "functionalizes"[All Fields] OR "functionalizing"[All Fields] OR "functionally"[All Fields] OR "functionals"[All Fields] OR "functioned"[All Fields] OR "functioning"[All Fields] OR "functionings"[All Fields] OR "functions"[All Fields] OR "physiology"[Subheading] OR "physiology"[All Fields] OR "function"[All Fields] OR "physiology"[MeSH Terms]  **Warnings**  (Autoimmune) AND ((Loss **of** function OR Loss-of-function) AND ((IFIH1) OR (MDA5 OR MDA-5))) |
| #10 | **#5 AND #8**  (IFIH1 OR MDA5 OR MDA-5) AND (Loss of function OR Loss-of-function) AND (inflammatory response)  19 results | ("inflammatories"[All Fields] OR "inflammatory"[All Fields]) AND ("response"[All Fields] OR "responses"[All Fields] OR "responsive"[All Fields] OR "responsiveness"[All Fields] OR "responsivenesses"[All Fields] OR "responsives"[All Fields] OR "responsivities"[All Fields] OR "responsivity"[All Fields]) AND ((("Loss"[All Fields] AND ("functional"[All Fields] OR "functional s"[All Fields] OR "functionalities"[All Fields] OR "functionality"[All Fields] OR "functionalization"[All Fields] OR "functionalizations"[All Fields] OR "functionalize"[All Fields] OR "functionalized"[All Fields] OR "functionalizes"[All Fields] OR "functionalizing"[All Fields] OR "functionally"[All Fields] OR "functionals"[All Fields] OR "functioned"[All Fields] OR "functioning"[All Fields] OR "functionings"[All Fields] OR "functions"[All Fields] OR "physiology"[MeSH Subheading] OR "physiology"[All Fields] OR "function"[All Fields] OR "physiology"[MeSH Terms])) OR "Loss-of-function"[All Fields]) AND ("IFIH1"[All Fields] OR ("MDA5"[All Fields] OR "MDA-5"[All Fields])))  **Translations**  **Inflammatory:** "inflammatories"[All Fields] OR "inflammatory"[All Fields]  **response:** "response"[All Fields] OR "responses"[All Fields] OR "responsive"[All Fields] OR "responsiveness"[All Fields] OR "responsivenesses"[All Fields] OR "responsives"[All Fields] OR "responsivities"[All Fields] OR "responsivity"[All Fields]  **function:** "functional"[All Fields] OR "functional's"[All Fields] OR "functionalities"[All Fields] OR "functionality"[All Fields] OR "functionalization"[All Fields] OR "functionalizations"[All Fields] OR "functionalize"[All Fields] OR "functionalized"[All Fields] OR "functionalizes"[All Fields] OR "functionalizing"[All Fields] OR "functionally"[All Fields] OR "functionals"[All Fields] OR "functioned"[All Fields] OR "functioning"[All Fields] OR "functionings"[All Fields] OR "functions"[All Fields] OR "physiology"[Subheading] OR "physiology"[All Fields] OR "function"[All Fields] OR "physiology"[MeSH Terms]  **Warnings**  (Inflammatory response) AND ((Loss **of** function OR Loss-of-function) AND ((IFIH1) OR (MDA5 OR MDA-5)))  **Stop word:** of |
| #11 | **#6 AND #8**  2 results | “SLE"[All Fields] AND ((("Loss"[All Fields] AND ("functional"[All Fields] OR "functional s"[All Fields] OR "functionalities"[All Fields] OR "functionality"[All Fields] OR "functionalization"[All Fields] OR "functionalizations"[All Fields] OR "functionalize"[All Fields] OR "functionalized"[All Fields] OR "functionalizes"[All Fields] OR "functionalizing"[All Fields] OR "functionally"[All Fields] OR "functionals"[All Fields] OR "functioned"[All Fields] OR "functioning"[All Fields] OR "functionings"[All Fields] OR "functions"[All Fields] OR "physiology"[MeSH Subheading] OR "physiology"[All Fields] OR "function"[All Fields] OR "physiology"[MeSH Terms])) OR "Loss-of-function"[All Fields]) AND ("IFIH1"[All Fields] OR ("MDA5"[All Fields] OR "MDA-5"[All Fields])))  **Translations**  **function:** "functional"[All Fields] OR "functional's"[All Fields] OR "functionalities"[All Fields] OR "functionality"[All Fields] OR "functionalization"[All Fields] OR "functionalizations"[All Fields] OR "functionalize"[All Fields] OR "functionalized"[All Fields] OR "functionalizes"[All Fields] OR "functionalizing"[All Fields] OR "functionally"[All Fields] OR "functionals"[All Fields] OR "functioned"[All Fields] OR "functioning"[All Fields] OR "functionings"[All Fields] OR "functions"[All Fields] OR "physiology"[Subheading] OR "physiology"[All Fields] OR "function"[All Fields] OR "physiology"[MeSH Terms]  **Warnings**  (SLE) AND ((Loss of function OR Loss-of-function) AND ((IFIH1) OR (MDA5 OR MDA-5)))  **Stop word:** of |
| #12 | **#8 OR #9 OR #10 OR #11**  169 results | ((("Loss"[All Fields] AND ("functional"[All Fields] OR "functional s"[All Fields] OR "functionalities"[All Fields] OR "functionality"[All Fields] OR "functionalization"[All Fields] OR "functionalizations"[All Fields] OR "functionalize"[All Fields] OR "functionalized"[All Fields] OR "functionalizes"[All Fields] OR "functionalizing"[All Fields] OR "functionally"[All Fields] OR "functionals"[All Fields] OR "functioned"[All Fields] OR "functioning"[All Fields] OR "functionings"[All Fields] OR "functions"[All Fields] OR "physiology"[MeSH Subheading] OR "physiology"[All Fields] OR "function"[All Fields] OR "physiology"[MeSH Terms])) OR "Loss-of-function"[All Fields]) AND ("IFIH1"[All Fields] OR ("MDA5"[All Fields] OR "MDA-5"[All Fields]))) OR (("autoimmune"[All Fields] OR "autoimmunity"[MeSH Terms] OR "autoimmunity"[All Fields] OR "autoimmunities"[All Fields] OR "autoimmunization"[All Fields] OR "autoimmunizing"[All Fields]) AND ((("Loss"[All Fields] AND ("functional"[All Fields] OR "functional s"[All Fields] OR "functionalities"[All Fields] OR "functionality"[All Fields] OR "functionalization"[All Fields] OR "functionalizations"[All Fields] OR "functionalize"[All Fields] OR "functionalized"[All Fields] OR "functionalizes"[All Fields] OR "functionalizing"[All Fields] OR "functionally"[All Fields] OR "functionals"[All Fields] OR "functioned"[All Fields] OR "functioning"[All Fields] OR "functionings"[All Fields] OR "functions"[All Fields] OR "physiology"[MeSH Subheading] OR "physiology"[All Fields] OR "function"[All Fields] OR "physiology"[MeSH Terms])) OR "Loss-of-function"[All Fields]) AND ("IFIH1"[All Fields] OR ("MDA5"[All Fields] OR "MDA-5"[All Fields])))) OR (("inflammatories"[All Fields] OR "inflammatory"[All Fields]) AND ("response"[All Fields] OR "responses"[All Fields] OR "responsive"[All Fields] OR "responsiveness"[All Fields] OR "responsivenesses"[All Fields] OR "responsives"[All Fields] OR "responsivities"[All Fields] OR "responsivity"[All Fields]) AND ((("Loss"[All Fields] AND ("functional"[All Fields] OR "functional s"[All Fields] OR "functionalities"[All Fields] OR "functionality"[All Fields] OR "functionalization"[All Fields] OR "functionalizations"[All Fields] OR "functionalize"[All Fields] OR "functionalized"[All Fields] OR "functionalizes"[All Fields] OR "functionalizing"[All Fields] OR "functionally"[All Fields] OR "functionals"[All Fields] OR "functioned"[All Fields] OR "functioning"[All Fields] OR "functionings"[All Fields] OR "functions"[All Fields] OR "physiology"[MeSH Subheading] OR "physiology"[All Fields] OR "function"[All Fields] OR "physiology"[MeSH Terms])) OR "Loss-of-function"[All Fields]) AND ("IFIH1"[All Fields] OR ("MDA5"[All Fields] OR "MDA-5"[All Fields])))) OR ("SLE"[All Fields] AND ((("Loss"[All Fields] AND ("functional"[All Fields] OR "functional s"[All Fields] OR "functionalities"[All Fields] OR "functionality"[All Fields] OR "functionalization"[All Fields] OR "functionalizations"[All Fields] OR "functionalize"[All Fields] OR "functionalized"[All Fields] OR "functionalizes"[All Fields] OR "functionalizing"[All Fields] OR "functionally"[All Fields] OR "functionals"[All Fields] OR "functioned"[All Fields] OR "functioning"[All Fields] OR "functionings"[All Fields] OR "functions"[All Fields] OR "physiology"[MeSH Subheading] OR "physiology"[All Fields] OR "function"[All Fields] OR "physiology"[MeSH Terms])) OR "Loss-of-function"[All Fields]) AND ("IFIH1"[All Fields] OR ("MDA5"[All Fields] OR "MDA-5"[All Fields]))))  **Translations**  **function:** "functional"[All Fields] OR "functional's"[All Fields] OR "functionalities"[All Fields] OR "functionality"[All Fields] OR "functionalization"[All Fields] OR "functionalizations"[All Fields] OR "functionalize"[All Fields] OR "functionalized"[All Fields] OR "functionalizes"[All Fields] OR "functionalizing"[All Fields] OR "functionally"[All Fields] OR "functionals"[All Fields] OR "functioned"[All Fields] OR "functioning"[All Fields] OR "functionings"[All Fields] OR "functions"[All Fields] OR "physiology"[Subheading] OR "physiology"[All Fields] OR "function"[All Fields] OR "physiology"[MeSH Terms]  **Autoimmune:** "autoimmune"[All Fields] OR "autoimmunity"[MeSH Terms] OR "autoimmunity"[All Fields] OR "autoimmunities"[All Fields] OR "autoimmunization"[All Fields] OR "autoimmunizing"[All Fields]  **function:** "functional"[All Fields] OR "functional's"[All Fields] OR "functionalities"[All Fields] OR "functionality"[All Fields] OR "functionalization"[All Fields] OR "functionalizations"[All Fields] OR "functionalize"[All Fields] OR "functionalized"[All Fields] OR "functionalizes"[All Fields] OR "functionalizing"[All Fields] OR "functionally"[All Fields] OR "functionals"[All Fields] OR "functioned"[All Fields] OR "functioning"[All Fields] OR "functionings"[All Fields] OR "functions"[All Fields] OR "physiology"[Subheading] OR "physiology"[All Fields] OR "function"[All Fields] OR "physiology"[MeSH Terms]  **Inflammatory:** "inflammatories"[All Fields] OR "inflammatory"[All Fields]  **response:** "response"[All Fields] OR "responses"[All Fields] OR "responsive"[All Fields] OR "responsiveness"[All Fields] OR "responsivenesses"[All Fields] OR "responsives"[All Fields] OR "responsivities"[All Fields] OR "responsivity"[All Fields]  **function:** "functional"[All Fields] OR "functional's"[All Fields] OR "functionalities"[All Fields] OR "functionality"[All Fields] OR "functionalization"[All Fields] OR "functionalizations"[All Fields] OR "functionalize"[All Fields] OR "functionalized"[All Fields] OR "functionalizes"[All Fields] OR "functionalizing"[All Fields] OR "functionally"[All Fields] OR "functionals"[All Fields] OR "functioned"[All Fields] OR "functioning"[All Fields] OR "functionings"[All Fields] OR "functions"[All Fields] OR "physiology"[Subheading] OR "physiology"[All Fields] OR "function"[All Fields] OR "physiology"[MeSH Terms]  **function:** "functional"[All Fields] OR "functional's"[All Fields] OR "functionalities"[All Fields] OR "functionality"[All Fields] OR "functionalization"[All Fields] OR "functionalizations"[All Fields] OR "functionalize"[All Fields] OR "functionalized"[All Fields] OR "functionalizes"[All Fields] OR "functionalizing"[All Fields] OR "functionally"[All Fields] OR "functionals"[All Fields] OR "functioned"[All Fields] OR "functioning"[All Fields] OR "functionings"[All Fields] OR "functions"[All Fields] OR "physiology"[Subheading] OR "physiology"[All Fields] OR "function"[All Fields] OR "physiology"[MeSH Terms]  **Warnings**  ((Loss **of** function OR Loss-of-function) AND ((IFIH1) OR (MDA5 OR MDA-5))) OR ((Autoimmune) AND ((Loss **of** function OR Loss-of-function) AND ((IFIH1) OR (MDA5 OR MDA-5)))) OR ((Inflammatory response) AND ((Loss **of** function OR Loss-of-function) AND ((IFIH1) OR (MDA5 OR MDA-5)))) OR ((SLE) AND ((Loss **of** function OR Loss-of-function) AND ((IFIH1) OR (MDA5 OR MDA-5))))  **Stop words:** of, of, of, of |

**Supplement Table 2: Search Strategy Medline (Ovid)** (09/03/24)**.**

| #1 | **IFIH1**  1778 results |  |
| --- | --- | --- |
| #2 | **MDA5 OR MDA-5**  2949 results | (MDA5 or MDA-5).mp. [mp=title, book title, abstract, original title, name of substance word, subject heading word, floating sub-heading word, keyword heading word, organism supplementary concept word, protocol supplementary concept word, rare disease supplementary concept word, unique identifier, synonyms, population supplementary concept word, anatomy supplementary concept word] |
| #3 | **Loss of function OR Loss-of-function**  57907 results | (loss of function or loss-of-function).mp. [mp=title, book title, abstract, original title, name of substance word, subject heading word, floating sub-heading word, keyword heading word, organism supplementary concept word, protocol supplementary concept word, rare disease supplementary concept word, unique identifier, synonyms, population supplementary concept word, anatomy supplementary concept word] |
| #4 | **Autoimmune**  239781 results |  |
| #5 | **Inflammatory response**  117513 results |  |
| #6 | **SLE**  44424 results |  |
| #7 | **#1 OR #2**  (IFIH1 OR MDA5 OR MDA-5)  3513 results |  |
| #8 | **#3 AND #7**  (IFIH1 OR MDA5 OR MDA-5) AND (Loss of function OR Loss-of-function)  35 results |  |
| #9 | **#4 AND #8**  (IFIH1 OR MDA5 OR MDA-5) AND (Loss of function OR Loss-of-function) AND (autoimmune)  10 results |  |
| #10 | **#5 AND #8**  (IFIH1 OR MDA5 OR MDA-5) AND (Loss of function OR Loss-of-function) AND (inflammatory response)  2 results |  |
| #11 | **#6 AND #8**  (IFIH1 OR MDA5 OR MDA-5) AND (Loss of function OR Loss-of-function) AND (SLE)  1 result |  |
| #12 | **#8 OR #9 OR #10 OR #11**  35 results |  |

**Supplement Table 3: Search Strategy PubMed** (07/02/2026).

| #1 | **Anti-MDA5 antibody**  772 results | ("anti-MDA5"[All Fields] AND ("antibodie"[All Fields] OR "antibodies"[Supplementary Concept] OR "antibodies"[All Fields] OR "antibodies"[MeSH Terms] OR "antibody s"[All Fields] OR "antibodys"[All Fields] OR "immunoglobulins"[Supplementary Concept] OR "immunoglobulins"[All Fields] OR "antibody"[All Fields] OR "immunoglobulins"[MeSH Terms])) AND (english[Filter])  **Translations**  **antibody:** "antibodie"[All Fields] OR "antibodies"[Supplementary Concept] OR "antibodies"[All Fields] OR "antibodies"[MeSH Terms] OR "antibody's"[All Fields] OR "antibodys"[All Fields] OR "immunoglobulins"[Supplementary Concept] OR "immunoglobulins"[All Fields] OR "antibody"[All Fields] OR "immunoglobulins"[MeSH Terms] |
| --- | --- | --- |
| #2 | **Anti-MDA5**  877 results | ("anti-MDA5"[All Fields]) AND (english[Filter]) |
| #3 | **COVID-19**  488,265 results | Search: **COVID-19** Filters: **English**  ("covid 19"[All Fields] OR "covid19"[All Fields] OR "covid 19"[MeSH Terms] OR "covid 19 vaccines"[All Fields] OR "covid 19 vaccines"[MeSH Terms] OR "covid 19 serotherapy"[All Fields] OR "covid 19 serotherapy"[MeSH Terms] OR "covid 19 nucleic acid testing"[All Fields] OR "covid 19 nucleic acid testing"[MeSH Terms] OR "covid 19 serological testing"[All Fields] OR "covid 19 serological testing"[MeSH Terms] OR "covid 19 testing"[All Fields] OR "covid 19 testing"[MeSH Terms] OR "sars cov 2"[All Fields] OR "sarscov2"[All Fields] OR "sarscov 2"[All Fields] OR "sars cov2"[All Fields] OR "sars cov 2"[MeSH Terms] OR "severe acute respiratory syndrome coronavirus 2"[All Fields] OR "2019 ncov"[All Fields] OR (("coronavirus"[MeSH Terms] OR "coronavirus"[All Fields] OR "cov"[All Fields] OR "ncov"[All Fields]) AND 2019/11/01:3000/12/31[Date - Publication])) AND (english[Filter])  **Translations**  **COVID-19:** ("COVID-19" OR "COVID19" OR "COVID-19"[MeSH Terms] OR "COVID-19 Vaccines" OR "COVID-19 Vaccines"[MeSH Terms] OR "COVID-19 serotherapy" OR "COVID-19 serotherapy"[MeSH Terms] OR "COVID-19 Nucleic Acid Testing" OR "covid-19 nucleic acid testing"[MeSH Terms] OR "COVID-19 Serological Testing" OR "covid-19 serological testing"[MeSH Terms] OR "COVID-19 Testing" OR "covid-19 testing"[MeSH Terms] OR "SARS-CoV-2" OR "SARSCoV2" OR "SARSCoV-2" OR "SARS-CoV2" OR "sars-cov-2"[MeSH Terms] OR "Severe Acute Respiratory Syndrome Coronavirus 2" OR "2019 NCOV" OR (("coronavirus"[MeSH Terms] OR "coronavirus" OR "COV" OR "NCOV") AND 2019/11/01[PDAT] : 3000/12/31[PDAT])) |
| #4 | **SARS-CoV-2**  260,973 results | ("sars cov 2"[MeSH Terms] OR "sars cov 2"[All Fields] OR "sars cov 2"[All Fields]) AND (english[Filter])  **Translations**  **SARS-COV-2:** "sars-cov-2"[MeSH Terms] OR "sars-cov-2"[All Fields] OR "sars cov 2"[All Fields] |
| # 5 | **Influenza**  149,984 results | Search: **influenza** Filters: **English**  ("influenza s"[All Fields] OR "influenza, human"[MeSH Terms] OR ("influenza"[All Fields] AND "human"[All Fields]) OR "human influenza"[All Fields] OR "influenza"[All Fields] OR "influenzas"[All Fields] OR "influenzae"[All Fields]) AND (english[Filter])  **Translations**  **influenza:** "influenza's"[All Fields] OR "influenza, human"[MeSH Terms] OR ("influenza"[All Fields] AND "human"[All Fields]) OR "human influenza"[All Fields] OR "influenza"[All Fields] OR "influenzas"[All Fields] OR "influenzae"[All Fields] |
| #6 | **Viruses**  1,474,349 results | Search: **viruses** Filters: **English**  ("virology"[MeSH Subheading] OR "virology"[All Fields] OR "viruses"[All Fields] OR "viruses"[MeSH Terms] OR "virus s"[All Fields] OR "viruse"[All Fields] OR "virus"[All Fields]) AND (english[Filter])  **Translations**  **viruses:** "virology"[Subheading] OR "virology"[All Fields] OR "viruses"[All Fields] OR "viruses"[MeSH Terms] OR "virus's"[All Fields] OR "viruse"[All Fields] OR "virus"[All Fields] |
| #7 | **#1 OR #2**  772 results |  |
| #8 | **#3 OR #4 OR #5 OR #6**  1,772,190 results |  |
| #9 | **#7 AND #8**  52 results |  |

**Supplement Table 4: *IFIH1* loss of function variants/MDA5 protein mutations**

| **c.DNA name** | **Protein Change** | **rsID** | **Effect on IFIH1/ MDA5 protein** | **Reference(s)** | **Associated conditions** |
| --- | --- | --- | --- | --- | --- |
| c.2016delA | p.Asp673fs  Position in protein: 672 out of 1025 | rs773033563  chromosome 2:162277443 | **Frameshift variant**  **Frameshift heterozygous variant. M**utation causing premature truncation; likely **loss of function (LoF)**. | (2, 11) | **IBD,** multisystem inflammatory syndrome in children (MIS-C), VEOIBD – complete MDA5 deficiency |
| c.1641+1G>C | **IVS8+1**  **Not assigned a standard p. (protein) notation** because it's a **splice-site variant**. | rs35337543  chromosome 2:16227995 | Splice donor variant  Disrupts essential splice site at intron 8 - likely exon skipping; LoF. | (2, 15, 17) (10, 16, 18) | Periodic fever, severe COVID-19, MIS-C, increased susceptibility to viral respiratory infections, T1DM association |
| c.2807+1G>A | p.Ile872Ter **IVS14+1** | rs35732034  chromosome 2:162268086 | **Splice donor variant**  **Splice site** mutation at donor site of exon 14; likely causes exon skipping or truncation; **LoF**. | (2, 11, 17, 19) (10, 15, 20) | **Severe COVID-19 showing immunodeficiency and glomerulonephritis, partial MDA5 deficiency VEOIBD (after neonatal period), increased susceptibility to viral respiratory infections, protection against hypothyroidism, T1D** |
| c.2665A>T | (p.Lys889Ter)  Position in protein: 889 out of 1025 | rs1252022173  chromosome 2: 162268229 | **Stop gained.**  **Nonsense** mutation resulting in early truncation of the C-terminal domain; **LoF**. | (17, 21) | **Increased susceptibility to infections** |
| c.1879G>T | (p.Glu627Ter)  Glu627X, E627X  Position in protein: 627 out of 1025 | rs35744605  chromosome 2:162277580 | **Stop gained.**  **Nonsense** mutation truncating the protein before the C-terminal domain; **LoF**. | (10, 11, 15, 17, 19-23) | **VEOIBD, increased susceptibility to viral respiratory infections, protections against hypothyroidism, protects against T1DM, IBD** |
| c.2836G>A | (Ala946Thr)  A946T (p.Ala946Thr)  Position in protein: 946 out of 1025 | rs1990760  chromosome 2:162267541 | Missense variant  Missense in helicase domain. | (10, 19, 23, 24) | lower risk of hypothyroidism, decreased risk of coronary artery disease, associated with human T1DM development, increased susceptibility to autoimmunity, increased susceptibility to chronic viral infections which can contribute to autoimmune responses in T1DM |
| c.2767A>G | I923V (p.Ile923Val)  Position in protein: 923 out of 1025 | rs35667974  chromosome 2:162268127 | Missense variant  Missense in C-terminal domain | (11, 15, 20, 22, 23) | Associated with ulcerative colitis (UC), protects against T1DM, IBD |
| c.2703C>A | p.Ile901Asn | Not assigned | **Missense** mutation in the helicase domain; uncertain effect, potentially alters RNA binding. | (2) | **Severe COVID-19** |
| c.1764del | p.Ala589fs  Position in protein: 686-588 out of 1025 | rs553669430  chromosome 2:162278213del | **Frameshift variant**  **Frameshift** mutation resulting in premature stop codon; likely **LoF**. | (18) | **N/A** |
| c.769+3A>G | p.Leu257fs | Not assigned | **Splice region** mutation near exon 8; likely disrupts normal splicing; potential **LoF**. | (18) | **N/A** |
| c.2035_2036delTT | p.Val679fs | Not assigned | **Frameshift** mutation leading to truncation; likely **LoF**. | (11) | **VEOIBD** |
| c.688C>T | p.Gln230Ter  Position in protein: 230 out of 1025 | rs771251917  chromosome 2:162306790 | **Stop gained**  **Nonsense** mutation leading to premature stop; likely results in **truncated, non-functional protein**. | (11) | **VEOIBD** |
| c.2044+2T>C |  | rs201026962 | **Splice donor variant** | (19) | **Protection against hypothyroidism** |
| c.454-1G>T |  | rs148590996  chromosome 2:162310934 | Splice acceptor variant  Disrupts **splice donor site** at intron 4; likely **exon skipping** or **frameshif**; **LoF.** | (19) | Protection against hypothyroidism |
| c.2528A>G | p.HIS843Arg  Position in protein: 843 out of 1025 | rs3747517  chromosome 2:162272314 | Missense variant | (10) | N/A |
| c.770-4185C.T |  | rs13023380  chromosome 1:162297853 | Intron variant | (10) | N/A |

*COVID-19: Coronavirus disease 2019,* ***IBD: inflammatory bowel disease,*** *LoF: loss-of-function, MIS-C: multisystem inflammatory syndrome in children,* ***N/A: not available,*** *T1DM: type 1 diabetes mellitus,* ***UC: ulcerating colitis.***
